# Adaptive Channel Estimation for Semi-Passive IRS with Optimized Sensor Deployment

**DOI:** 10.3390/s25216797

**Published:** 2025-11-06

**Authors:** Zhiyu Han, Hanning Wang, Yafeng Wang, Zhuo Fan

**Affiliations:** 1 The Key Laboratory of Universal Wireless Communications, Ministry of Education, Beijing University of Posts and Telecommunications, Beijing 100876, China; hanzhiyu@bupt.edu.cn (Z.H.); 2021010037@bupt.cn (Z.F.); 2 The Future Research Laboratory, China Mobile Research Institute, Beijing 100053, China; wanghanning@chinamobile.com

**Keywords:** Intelligent Reflecting Surface (IRS), channel estimation, compressed sensing, particle swarm optimization (PSO)

## Abstract

To achieve optimal passive beamforming gains from Intelligent Reflective Surfaces (IRS), accurate Channel State Information (CSI) acquisition is required. However, the IRS, with numerous passive devices, lacks the ability to process signals, resulting in considerable challenges in obtaining accurate CSI. Based on the semi-passive IRS, this paper proposes a compressed sensing channel estimation algorithm without knowing the path number of channel, which improves the accuracy of channel estimation. Furthermore, a particle swarm optimization (PSO)-based deployment scheme for active sensors in the semi-passive IRS is developed. Numerical simulations confirm the effectiveness, demonstrating a reduction in Normalized Mean Square Error (NMSE) and improved channel estimation with fewer pilot symbols, thereby minimizing estimation overhead.

## 1. Introduction

In recent years, the Intelligent Reflecting Surface (IRS) has received extensive attention from the engineering and academic communities owing to its property of enabling the channel environment to become autonomous and controllable [1,2,3]. The IRS is a panel consisting of numerous configurable electromagnetic elements [4,5]. By controlling the elements of the IRS, the physical characteristics of the electromagnetic elements can be dynamically adjusted, thus facilitating the reflection of signals incident on the IRS in the target direction. In addition, the IRS is cost-effective and facile to deploy, giving it the potential to be used to meet the requirements of creating future wireless mobile communication networks [6,7,8].

In practical applications, obtaining accurate channel state information (CSI) is essential to acquire an ideal IRS reflection matrix [9]. However, obtaining CSI poses several difficulties [10,11,12]. First, the IRS is composed of a large number of passive elements, which merely reflect the signal and lack the ability to receive as well as process the pilot symbol [13]. Second, in IRS-assisted wireless systems, because there are a great deal of elements in the IRS, numerous channel parameters need estimation, which leads to significant computational overhead for channel estimation [7]. Therefore, the algorithm design with the aim to achieve more accurate CSI is crucial for enhancing the performance of IRS-assisted wireless communication systems.

In [14,15], traditional channel estimation techniques such as the least square (LS) estimation and minimum mean square error (MMSE) estimation are applied to IRS-assisted wireless communication systems. These techniques are employed to estimate the cascade channel reflected by IRS, by switching off some passive elements of IRS at each time. However, the problem of high training overhead due to the necessary estimation of numerous channel parameters has not been effectively tackled. In addition, the algorithm does not acquire separate CSI. To address this issue, a semi-passive architecture IRS is proposed. It aims to replace the passive reflection elements of part of the IRS with active sensors with signal processing capability. Based on this, a deep denoising neural network assisted channel estimation for millimetre wave (mmWave) IRS systems was proposed in [16,17] to reduce the training overhead. In [18,19], the authors have exploited the sparsity of mmWave for channel estimation using the sparse Bayesian learning (SBL) approach and compressed sensing, respectively. However, in [16,18,19], a randomized layout of active sensors has been adopted, and the effect of the position of active sensors on the channel estimation results has yet to be investigated. Furthermore, the number of iterations of a compressed sensing signal recovery algorithm is usually related to the number of channel paths, which is difficult to obtain [20]. To tackle this problem, several adaptive compressed sensing algorithms have been developed to improve reconstruction performance by adjusting the measurement process or sparsity level dynamically. For instance, the sparsity adaptive matching pursuit (SAMP) [21] and block sparsity adaptive matching pursuit (BSAMP) [22] adaptively determine the support set size based on residual variations, while adaptive OMP variants modify atom selection rules according to the residual energy evolution [23]. These methods have demonstrated improved convergence and reconstruction accuracy in conventional mmWave/MIMO systems. However, in IRS-assisted channel estimation scenarios, the actual sparsity is often difficult to determine in advance, which may lead to performance degradation when fixed are used.

Building upon the above research, this paper investigates a semi-passive IRS-assisted millimeter-wave (mmWave) system. An adaptive channel estimation based on compressed sensing is proposed, eliminating the requirement to know the number of channel paths in advance, which is challenging to obtain in practical engineering. The impact of the deployment location of active sensors on channel estimation under the semi-passive IRS architecture is also investigated, and a deployment scheme based on particle swarm optimization (PSO) for placing the active sensors is developed. Simulation results show that the proposed scheme in this paper can effectively improve channel estimation accuracy and training overhead of channel estimation.

## 2. System Model and Transmission Scheme

### 2.1. System and Channel Model

**Figure 1:**
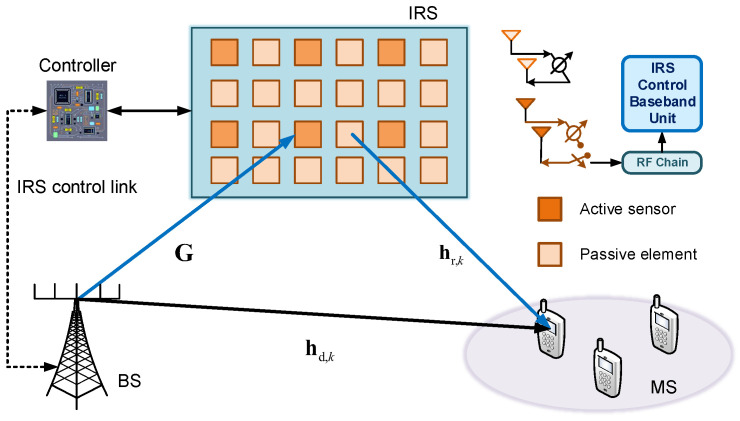
An semi-passive IRS-aided multi-user wireless communication system.

Figure 1 shows an IRS-assisted mmWave system, where the base station (BS) has a uniform linear array (ULA) of *M* antennas, with a half-wave length separation between each antenna. The IRS is a programmable metasurface made up of *N* elements, which is arranged in form of an $N_{1}\times N_{2}$ uniform planar array (UPA) with $N_{1}\times N_{2}=N$. In addition, there is *K* mobile stations (MS) with a single antenna. To mitigate the complexity associated with channel estimation, this paper proposes the replacement of certain passive elements within the IRS with $N_{A}$ active sensors equipped with channel sensing and signal processing capabilities. Such active sensors are equipped with radio frequency (RF) chains and mode-switching circuits. At the phase of channel estimation, it can receive and process the pilot symbols. At the phase of data transmission, it only reflects the signal like other passive elements.

In this paper, we consider a downlink communication scenario. The signal is transmitted by the BS and reaches the *k*th MS through two links. One is the direct channel from BS to the *k*th MS, $h_{d,k}^{H}\in C^{1\times M}$, that can be estimated using conventional channel estimation algorithms under deactivating the IRS. The other is the reflective link called cascaded transmit channel, which is divided into two parts, including the BS-IRS channel $G\in C^{N\times M}$ and the IRS-MS channel $h_{r,k}^{H}\in C^{1\times N}$. Then, the receive signal *y* at the *k*th MS could be expressed as

(1) $$y_{k}=\left(h_{r,k}^{H}\Omega G+h_{d,k}^{H}\right)x+n_{k},$$

where $x\in C^{M}$ denotes the transmitted signal from BS and $n_{k}∼CN\left(0,\sigma^{2}\right)$ denotes the additive noise, $\Omega \in C^{N\times N}$ is reflection matrix of IRS.

The BS-IRS channel can be modeled via the Saleh-Valenzuela channel model [24] as

(2) $$G=\sqrt{\frac{MN}{L}}\sum_{l=1}^{L}\beta_{l}a_{I}\left(\psi_{l},\varphi_{l}\right)a_{B}^{H}\left(\theta_{l}\right),$$

where *L* represents the path number of BS-IRS channel. $\beta_{l}$ represents the path-loss coefficient and $\theta_{l}$ is the angle of departure (AOD) at BS, $\psi_{l}$ and $\varphi_{l}$ are the azimuth and elevation angle of arrival (AOA) at IRS. Furthermore, the array response vector $a_{B}\left(\theta_{l}\right)$ at BS can be formulated as

(3) $$a_{B}\left(\theta_{l}\right)=\frac{1}{\sqrt{M}}{\left[1,e^{j\frac{2\pi d}{\lambda }\sin\theta_{l}},\ldots ,e^{j\frac{2\pi d}{\lambda }\left(M-1\right)\sin\theta_{l}}\right]}^{T},$$

where $d=\frac{\lambda }{2}$ and λ is the carrier wavelength. The array response matrix $a_{I}\left(\psi_{l},\varphi_{l}\right)$ at IRS is

(4) $$a_{I}\left(\psi_{l},\varphi_{l}\right)=a_{I,v}^{l}⊗a_{I,h}^{l},$$

where

(5) $$a_{I,v}^{l}=\frac{1}{\sqrt{N_{1}}}{\left[1,e^{j\frac{2\pi d}{\lambda }\sin\psi_{l}},\ldots ,e^{j\frac{2\pi d}{\lambda }\left(N_{1}-1\right)\sin\psi_{l}}\right]}^{T},$$

(6) $$a_{I,h}^{l}=\;\frac{1}{\sqrt{N_{2}}}{\left[1,\;e^{\;j\;\frac{2\pi d}{\lambda }cos\varphi_{l}\;\sin\;\psi_{l}},\;\ldots \;,e^{\;j\;\frac{2\pi d}{\lambda }\;\left(N_{2}-1\right)cos\varphi_{l}\;\sin\;\psi_{l}}\right]}^{T}.$$

Similarly, the channel between IRS and the *k*th MS can be formulated as

(7) $$h_{r,k}^{H}=\sum_{l^{\prime }=1}^{L_{\mathrm{MS},k}}\beta_{l^{\prime },k}^{\mathrm{MS}}a_{I}^{H}\left(\psi_{l^{\prime },k}^{\mathrm{MS}},\varphi_{l^{\prime },k}^{\mathrm{MS}}\right),$$

where $L_{\mathrm{MS},k}$ is the path number of IRS-MS channel.

The BS-IRS channel as in (2) can be redefined in the following more compact form:

(8) $$G=A_{I}D_{\beta }A_{B}^{H},$$

where $A_{I}=\left[a_{I}\left(\psi_{1},\varphi_{1}\right),\ldots ,a_{I}\left(\psi_{L},\varphi_{L}\right)\right]$; $A_{B}=\left[a_{B}\left(\theta_{1}\right),\ldots ,a_{B}\left(\theta_{L}\right)\right]$; $\left[\beta_{1},\ldots ,\beta_{L}\right]$ is positioned on the diagonal of the square matrix $D_{\beta }\in C^{L\times L}$.

Since the channel estimation method does not know $A_{I}$ and $A_{B}$, we will employ the virtual channel form to rebuild (8) as

(9) $$G=V_{I}G_{V}V_{B}^{H},$$

where the unitary discrete Fourier transform (DFT) matrices of size N×N and M×M, respectively, are denoted by $V_{I}$ and $V_{B}$. $G_{V}$ represents the dimension N×M of the virtual channel element matrix.

### 2.2. Transmission Scheme

**Figure 2:**
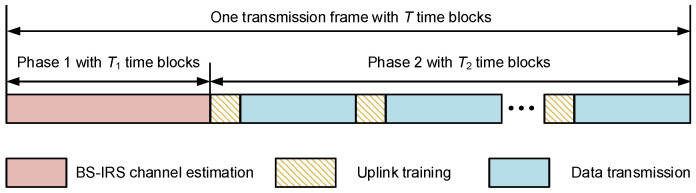
Proposed transmission protocol.

In this paper, we consider a time-division duplexing (TDD) system and assume that the BS–IRS channel remains constant within each transmission frame. The transmission frame under consideration comprises *T* time blocks, each containing τ time slots/symbols, as illustrated in Figure 2. During a time block, the IRS-MS channel is static.

Two phases can be separated in a transmission frame. The first phase consists of $T_{1}$ time blocks in which the BS sends the pilot symbols to the IRS. After that the active sensors of the IRS receives the pilot symbols, performs channel estimation and transmits its result to the BS via a feedback link. The second phase consist of $T_{2}=T-T_{1}$ time blocks. Within each time block, there is a subdivision into two sub-blocks, allocated for uplink channel estimation and downlink data transmission, respectively. In the sub-block of uplink channel estimation, the MS sends the pilot symbols to the IRS, and the active sensors in the IRS will receive the pilot symbols from the MS to perform the channel estimation, then the channel estimation result will be transmitted via the control link back to the BS. According to the TDD channel reciprocity, the BS can obtain the CSI of the downlink channel at the same time. Finally, the BS computes the reflection matrix based on the full CSI obtained and transmits it to the IRS. In the data transmission sub-block, the IRS switches to reflection mode and reflects the signal sent by the BS to the target MS.

## 3. Adaptive Channel Estimation Algorithm Based on Compressed Sensing

This section proposes an adaptive compressed sensing-based channel estimation (ACSCE) algorithm that aims to estimate the CSI of the BS-IRS channel G and the RIS-MS channel $h_{r,k}$, respectively. The algorithm is developed based on the semi-passive IRS architecture and does not need to be informed about the path number of the channel beforehand.

In this paper, we consider the BS-IRS channel as a representative scenario to illustrate the proposed estimation approach. The estimation method for IRS-MS channel will be the same as the BS-IRS channel.

In the channel estimation $T_{1}$ phase, the IRS receives the pilot symbols sent by the BS as

(10) $$Y_{\mathrm{BI}}=WGX_{P}+N,$$

where $X_{P}\in C^{M\times X_{L}}$ denotes the training signal from BS, with $X_{L}$ representing its length; $N\in C^{N_{A}\times X_{L}}$ denotes the additive noise; W is an $N_{A}\times N$ selection matrix, which serves to extract the entries from the original channel matrix G associated with the active sensors of the IRS. Mark by A the set of indices that relate to the active sensors of the IRS, with $\left|A\right|\;=\;N_{A}$. Then, the matrix $W={\left[I\right]}_{A,:}$, implying that W encompasses the rows of the N×N identity matrix I. The vectorized desired received signal can be represented as follows using the Kronecker product property:

(11) $$\begin{array}{cc} vec\left(Y_{\mathrm{BI}}\right)={\bar{y}}_{\mathrm{BI}} & =vec\left(WGX_{P}\right)+vec\left(N\right)\\ \; & =vec\left(WV_{I}G_{V}V_{B}^{H}X_{P}\right)+vec\left(N\right)\\ \; & ={\left(V_{B}^{H}X_{P}\right)}^{T}⊗\left(WV_{I}\right)g_{s}+vec\left(N\right)\\ \; & =\Theta g_{s}+vec\left(N\right), \end{array}$$

where $g_{s}$ denotes the vectorized form of the $G_{V}$, $\Theta ={\left(V_{B}^{H}X_{P}\right)}^{T}⊗\left(WV_{I}\right)$ denotes the equivalent sensing matrix. Now, we give the received signal vector of BS-IRS link ${\bar{y}}_{\mathrm{BI}}$ and the sensing matrix Θ. To estimate the sparse vector $g_{s}$, solving the non-convex combination problem is required.

(12) $$\min{∥g_{s}∥}_{0}\;s.t.∥{\bar{y}}_{\mathrm{BI}}-\Theta g_{s}∥\leq \xi .$$

To estimate the approximate solution $g_{s}$ based on the sparse formulation in (12), we propose improvements to the Orthogonal Matching Pursuit (OMP) algorithm in this paper. These improvements involve dynamically adjusting the number of iterations based on the channel environment, meeting the requirements of practical engineering scenarios. In each iteration, the algorithm selects the most correlated atom from the overcomplete dictionary to update the support set. Then, it constructs a sparse approximation to obtain the current estimate. The residual is updated by decomposing the signal components and computing the difference between the current and previous residuals. If the residual difference is less than the threshold ε, the iteration is halted; otherwise, the current residual is forwarded to the next iteration. The details of the proposed channel estimation algorithm are outlined in Algorithm 1.

Next, we analyze the computational complexity of the proposed algorithm. The computational complexity of the proposed algorithm mainly depends on the number of iterations and the size of the sensing matrix Θ. At each iteration, the major operations include correlation computation and LS estimation, which have similar computational order to those in the conventional OMP algorithm. Therefore, the overall complexity can be approximated as $O(QX_{L}N_{A}MN)$, where *Q* denotes the number of iterations.

The residual vector $r_{q}$ is updated through an orthogonal projection onto the complement of the subspace spanned by the selected atoms at each iteration. As a result, the residual energy $|r_{q}{|}_{2}^{2}$ monotonically decreases with *q*, i.e., $|r_{q+1}{|}_{2}^{2}\leq {\left|r_{q}\right|}_{2}^{2}$, and it is lower bounded by zero. Therefore, the algorithm guarantees monotonic convergence of the residual sequence.
**Algorithm 1** A semi-passive IRS-based channel estimation algorithm with adaptive capability**Require:** 
The sensing matrix Θ, observation vector ${\bar{y}}_{\mathrm{BI}}$.**Ensure:** 
The estimated sparse vector ${\hat{g}}_{s}$.  1: **Initialize** Residuals $r_{0}={\bar{y}}_{\mathrm{BI}}$, support set $\Lambda_{0}=\phi$, the number of iterations t=0, $\left[M,N\right]=\mathrm{size}\left(\Theta \right)$, the difference of residuals $\Delta_{0}=∥r_{0}{∥}_{2}/M$, threshold value ε.  2: **while** $\Delta_{q}>\varepsilon$ **do**  3:  q=q+1.  4:  $s_{q}=\arg\max\left({∥\Theta^{T}r_{q-1}∥}_{2}\right)$.    //Find the index of the column with the most relevant residuals.  5:  $\Delta_{q}=\Delta_{q-1}\cup s_{q}$.    //Store column indexes and expand support sets.  6:  ${\hat{g}}_{q}={\left(\Theta_{\Lambda_{t}}^{T}\Theta_{\Lambda_{q}}\right)}^{-1}\Theta_{\Lambda_{q}}^{T}{\bar{y}}_{\mathrm{BI}}$. //least squares solution.  7:  $r_{q}={\bar{y}}_{\mathrm{BI}}-\Theta_{\Lambda_{q}}{\hat{g}}_{q}={\bar{y}}_{\mathrm{BI}}-\Theta_{\Lambda_{q}}{\left(\Theta_{\Lambda_{q}}^{T}\Theta_{\Lambda_{q}}\right)}^{-1}\Theta_{\Lambda_{q}}^{T}{\bar{y}}_{\mathrm{BI}}$.    //Calculate residuals.  8:  $\Theta \left(:,s_{q}\right)=zeros\left(M,s_{q}\right)$.    //Orthogonalization process.  9:  $\Delta_{q}=abs\left({∥r_{q}∥}_{2}-{∥r_{q-1}∥}_{2}\right)/N$.    //Calculate the difference of the residuals.10: **end while**11: ${\hat{g}}_{s}={\hat{g}}_{q}$, ${\hat{g}}_{\Delta_{q}^{c}}=0$.   //$\Delta_{q}$ and $\Delta_{q}^{c}$ are a set of complementary indices.12: **return** ${\hat{g}}_{s}$.

## 4. PSO-Based Deployment Optimization of IRS Active Sensors Assisted by the ACSCE Algorithm

In this section, building upon the adaptive channel estimation algorithm proposed in Section 3, we further develop a PSO-based deployment scheme for IRS active sensors (PIAS). By improving the PSO algorithm [25], the proposed method utilizes the channel estimation NMSE as the optimization objective, enabling the IRS to automatically determine the optimal placement of active sensors for enhanced channel estimation performance.

Consider an IRS consisting of *N* reflection elements with $N_{A}$ active sensors. Initially, each reflection element of the IRS is numbered, and $p_{n_{A}}$ denotes the location of the $n_{A}$th active sensor. Assume that the result of the BS-IRS channel estimation is denoted by $\hat{G}$. The optimization objective is to reduce the NMSE of the channel estimation.

For the IRS in this paper, the optimization constraints include the size of the panel as well as the spacing of adjacent active sensors. Therefore, this optimization model under multiple constraints can be treated as seeking a set of optimal array index intervals vectors $d=\left[d_{1},d_{2},\ldots ,d_{N_{A}-1}\right]$ to minimize the NMSE of the channel estimation, which is described as follows:

(13) $$\begin{array}{cc} \min\; & NMSE=10\log_{10}\frac{E\left\{{∥\hat{G}-G∥}_{F}^{2}\right\}}{E\left\{{∥G∥}_{F}^{2}\right\}}, \end{array}$$

(14) $$\begin{array}{cc} s.t.\; & d_{n_{A}}\geq d_{\min},1\leq n_{A}\leq N_{A}-1, \end{array}$$

(15) $$\begin{array}{cc} & \sum_{n_{A}=1}^{N-1}d_{n_{A}}=N, \end{array}$$

where $d_{n_{A}}=p_{n_{A}+1}-p_{n_{A}}$ denotes the active sensors index interval and $d_{\min}$ denotes the minimum index interval constraint of neighboring active sensors.

Assuming a particle swarm $P=\left\{p_{1},p_{2},\ldots ,p_{J}\right\}$ consisting of *J* particles, each particle position $p_{j}=\left[p_{j1},p_{j2},\ldots ,p_{jN_{A}}\right]$ corresponds to a potentially feasible solution of the optimization problem, where $p_{jn_{A}}$ denotes the location of the $n_{A}$th active sensors in the *j*th particle, the velocity of each particle is $v_{j}=\left[v_{j1},v_{j2},\ldots ,v_{jN_{A}}\right]$. During each iteration, the particle is subjected to the global optimal solution and the local optimal solution of the optimization search process, denoted by $p_{g}$ and $p_{l}$, respectively. Then, the velocity and position of the particle are updated according to the following notation:

(16) $$v_{j}\left(t+1\right)=\omega v_{j}\left(t\right)+c_{1}r_{1}\left[p_{l}-p_{j}\left(t\right)\right]-c_{2}r_{2}\left[p_{g}-p_{j}\left(t\right)\right],$$

(17) $$p_{j}\left(t+1\right)=p_{j}\left(t\right)+v_{j}\left(t+1\right),$$

where ω represents inertia terms; $c_{1}$ and $c_{2}$ are acceleration coefficients; $r_{1}$ and $r_{2}$ are random numbers in the interval $\left[0,1\right]$.

In order to prevent active sensors from going beyond the IRS range, we will set up two virtual active sensors, specifying their coordinate as 0 and N+1. Taking the minimum index interval between two selected active sensors as $d_{\min}=1$, the total intervals occupied by $N_{A}$ active sensors and two virtual active sensors is $\left(N_{A}+1\right)d_{\min}$, so the remaining interval of the panel that can be used for non-uniform arrangement is $S=\left(N+1\right)-\left(N_{A}+1\right)d_{\min}$. In this closed interval $\left[0,S\right)$, generate $N_{A}$ random numbers and sort them from smallest to largest to get the initial particle swarm position $s=\left[s_{1},s_{2},\ldots ,s_{N_{A}}\right]$. By adding the spacing constraint vector d, the vector consisting of $N_{A}$ reflective cells in the panel is obtained as $s+d=\left[s_{1},s_{2},\ldots ,s_{N_{A}}\right]+\left[d_{\min},2d_{\min},\ldots ,N_{A}d_{\min}\right]$. A complete vector of array elements including $N_{A}+2$ individual cells is $p=\left[p_{0},p_{1},p_{2},\ldots ,p_{N_{A}},p_{N_{A}+1}\right]=\left[0,s_{1}+d_{\min},s_{2}+2d_{\min},\ldots ,s_{N_{A}}+N_{A}d_{\min},N+1\right]$. Obviously, the individual generated by the above method satisfies the constraints that the number of active sensors is $N_{A}$, the panel size is *N*, and the minimum reflective cell spacing constraint is $d_{\min}$. The optimization with individual constraints is reduced to an unconstrained optimization problem.

The PSO algorithm for optimal layout is implemented in Algorithm 2.
**Algorithm 2** PSO-based deployment scheme for IRS active sensors**Require:** 
the number of the active sensors $N_{A}$, the number of the IRS reflection elements *N*.**Ensure:** 
The selection matrix W.  1: **Initialize** the number of particle swarm *J*, the spacing constraints of adjacent active sensors $d_{\min}$, the inertia terms ω, the acceleration coefficients $c_{1}$ and $c_{2}$, the velocity of each particle $v_{j}\left(0\right)=zeros\left(1,N_{A}\right)$, the number of iterations t=0, the maximum number of iterations $t_{\max}$.  2: **while** $t\leq t_{\max}$ **do**  3:  t=t+1.  4:  Randomly initialize the particle positions based on the particle swimming range determined by the IRS.  5:  Calculate the initial fitness function value by (13).  6:  Every particle has its position and velocity updated according to (16) and (17).  7:  Solve for the fitness value of all particles.  8: **end while**  9: Set $A=\left\{p_{n_{A}}^{*}\;=\;\left\lfloor p_{n_{A}}\right\rfloor \left|p_{n_{A}}\;=\;{\left[p_{g}\right]}_{n_{A},1},n_{A}=1,\ldots ,N_{A}\right.\right\}$.10: $W={\left[I\right]}_{A,:}$. 11: **return** W.

## 5. Numerical Results

To assess the validity of our suggested schemes and methodology, we carried a numerical simulation in this section. In this paper, we consider an IRS-assisted mmWave system, where the number of BS antennas and IRS elements are respectively M=8 and N=64. Moreover, the IRS is equipped with $N_{A}=16$ active sensors that are utilized to estimate the channel for the BS-IRS. The path number of BS-IRS link is L=3, and the path number of IRS-MS link is $L_{MS,k}=1$ for ∀k. The path-loss coefficient is assumed to follow a complex Gaussian distribution with unit power [26]. $\psi_{l}$, $\varphi_{l}$, $\theta_{l}$, $\psi_{l^{\prime },k}^{\mathrm{MS}}$ and $\varphi_{l^{\prime },k}^{\mathrm{MS}}$ are continuous and uniformly distributed over $\left[-\frac{\pi }{2},\frac{\pi }{2}\right)$. The performance metric chosen is the NMSE.

**Figure 3:**
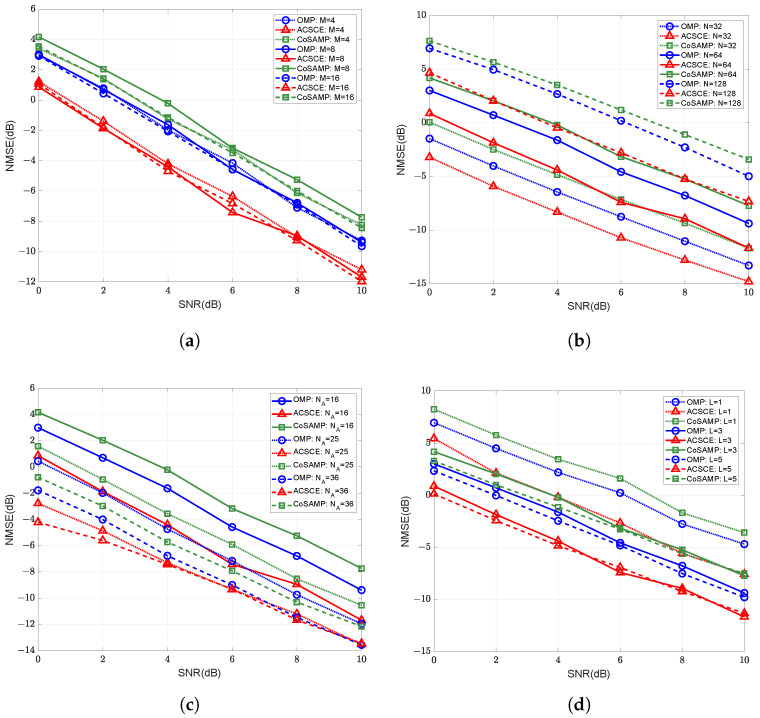
The channel estimation NMSE versus SNR with different compressed sensing algorithms under (**a**) varying numbers of BS antennas, (**b**) varying numbers of IRS elements, (**c**) varying numbers of active sensors, (**d**) varying numbers of BS-IRS paths.

Figure 3 displays the comparison between the proposed ACSCE algorithm with conventional compressed sensing approaches in terms of NMSE performance under various configurations. Among these, the algorithms employ the same active sensor configuration. We fix all other parameters and vary only one parameter to investigate the algorithm’s performance. Figure 3 reveals that the algorithm maintains superior channel estimation performance across different antenna configurations, IRS sizes, numbers of active sensors, and path counts, particularly under low SNR. Compared to other traditional channel estimation algorithms, it demonstrates significant performance improvements. Specifically, under the conditions M=8, N=64, $N_{A}=16$, and the path count of BS-IRS L=3, the NMSE of the ACSCE algorithm in this paper decreases by 2.57 dB and 3.91 dB on average compared with the OMP [19] and CoSAMP [27], respectively. This indicates that the proposed ACSCE algorithm possesses robustness and scalability. Moreover, as illustrated in Figure 3c, increasing the number of active sensors further improves the channel estimation accuracy. This demonstrates that an improve channel estimation accuracy can be achieved at the cost of only a slight increase in hardware complexity.

**Figure 4:**
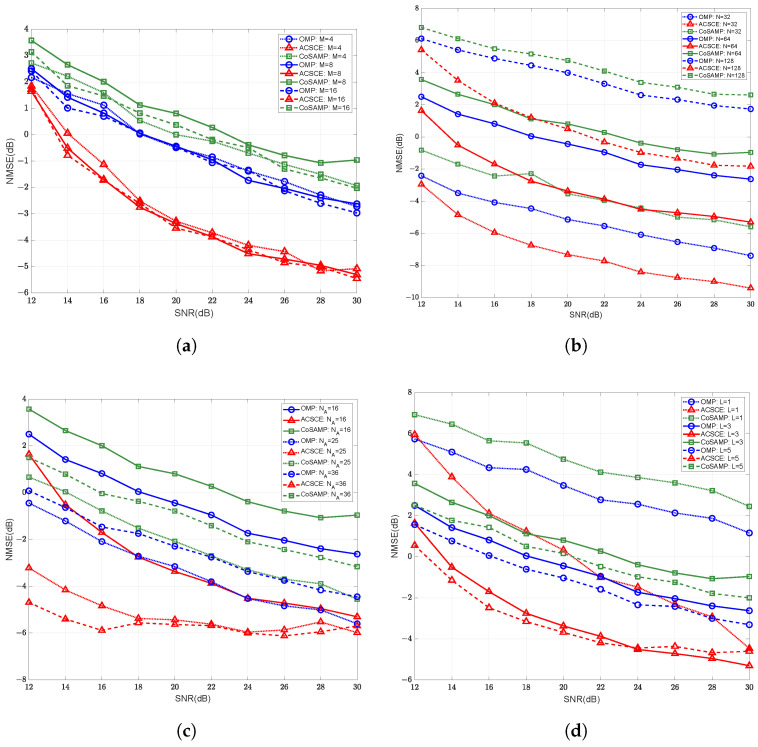
The channel estimation NMSE versus the number of pilots with different compressed sensing algorithms under: (**a**) varying numbers of BS Antennas, (**b**) varying numbers of IRS elements, (**c**) varying numbers of active sensors, (**d**) varying number of BS-IRS paths.

Figure 4 illustrates the NMSE performance of the proposed ACSCE algorithm in comparison with conventional compressed sensing methods under varying pilot lengths. Among these, the algorithms employ identical active sensor configuration and have SNR of 2dB. All other parameters were kept constant, while only one parameter was varied to evaluate the algorithm’s performance under different pilot lengths. Figure 4 shows that the proposed algorithm consistently achieves superior channel estimation performance across different antenna configurations, IRS sizes, numbers of active sensing devices, and path counts. For the same NMSE level, the ACSCE algorithm requires the shortest pilot length. Specifically, when M=8, N=64, $N_{A}=16$, and the number of paths is 3, the proposed ACSCE algorithm achieves an average pilot overhead reduction of about 25%. Figure 4c presents the NMSE performance under different numbers of active sensors with varying pilot lengths. It can be seen that increasing the number of active sensors significantly reduces the required pilot length for achieving the same NMSE. This demonstrates that with only a slight increase in hardware complexity, the proposed system can achieve higher estimation accuracy with lower training overhead. This further reduces the time required for channel estimation.

**Figure 5:**
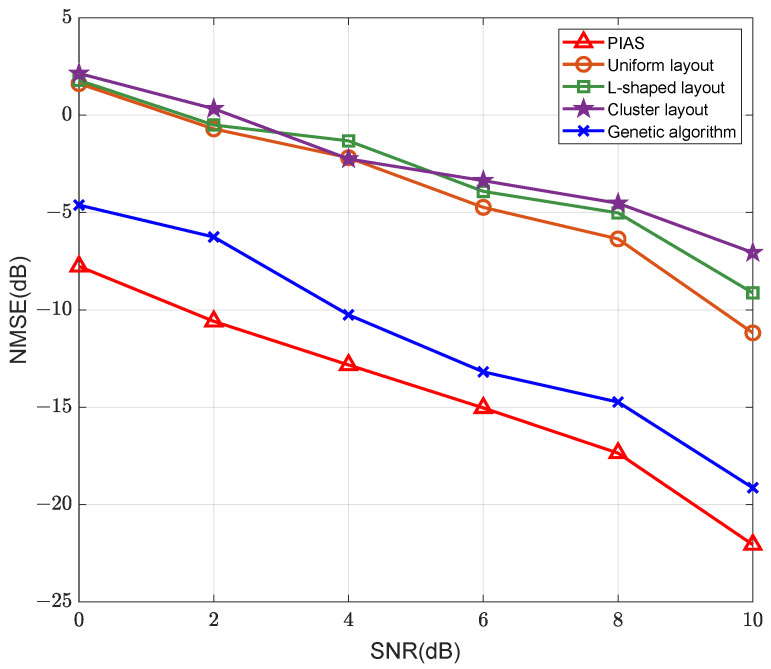
NMSE versus the number of iterations for IRS channel estimation under different active sensor deployment scheme.

Figure 5 presents the channel estimation performance of the proposed algorithm under various active sensor deployment strategies. The simulation results indicate that different active sensors deployment schemes have a large impact on the channel estimation. The channel estimation performance using the deployment scheme achieves superior performance compared with uniform layout, clustered layout [28], L-shaped layout [29], and genetic algorithm [30], yielding a noticeable enhancement in estimation accuracy.

**Figure 6:**
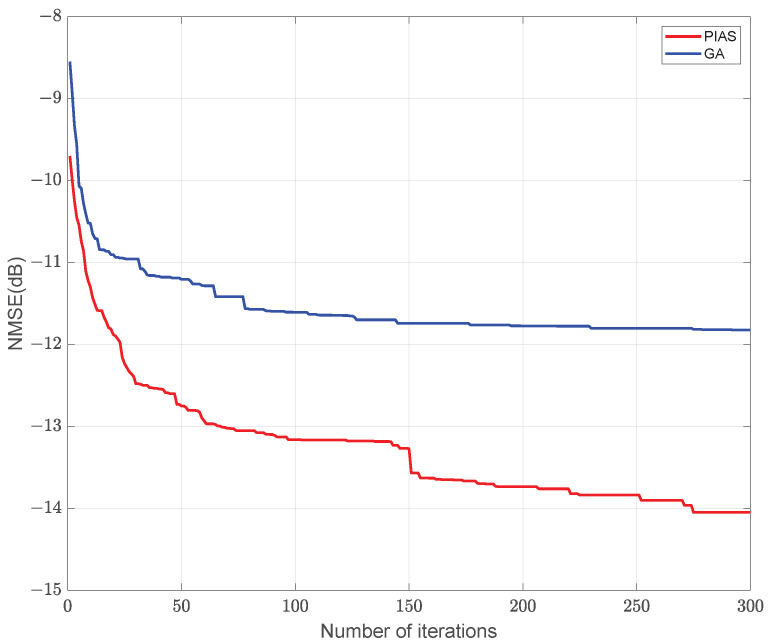
Convergence curves under different IRS active sensor deployment schemes.

Figure 6 illustrates the NMSE performance of channel estimation versus the number of iterations under various optimization deployment strategies for active sensing devices in semi-passive IRS systems. As observed in Figure 6, the proposed PIAS algorithm achieves a lower NMSE than the genetic algorithm for the same number of iterations. This result demonstrates that the proposed algorithm exhibits effective convergence and improved channel estimation accuracy.

## 6. Conclusions

This paper investigated channel estimation for IRS-assisted mmWave systems by jointly optimizing the IRS hardware architecture and the estimation algorithm. An adaptive compressed sensing-based channel estimation algorithm was proposed, which reconstructs the cascaded channel without prior knowledge of path parameters. Moreover, a PSO-based semi-passive IRS deployment scheme was designed to determine the optimal positions of active sensors. Simulation results confirmed the effectiveness of the proposed framework, achieving approximately 25% reduction in pilot overhead while maintaining comparable estimation accuracy. The PSO-based deployment further yielded a notable NMSE improvement (up to 2.5 dB) compared with random or uniform configurations. In future work, the proposed algorithm could be extended to beam-scanning antenna array designs, where sparse reconstruction can facilitate direction estimation and beam calibration. Integrating this algorithm into adaptive beamforming architectures [31,32] is expected to enhance scanning adaptability and estimation robustness, demonstrating the potential of the proposed method for intelligent reconfigurable array systems.

## Data Availability

Data are contained within the article.
